# *GJA1* Gene Polymorphisms and Topographic Distribution of Cranial MRI Lesions in Cerebral Small Vessel Disease

**DOI:** 10.3389/fneur.2020.583974

**Published:** 2020-11-25

**Authors:** Jing Zhang, Qian You, Junlong Shu, Qiang Gang, Haiqiang Jin, Meng Yu, Wei Sun, Wei Zhang, Yining Huang

**Affiliations:** Department of Neurology, Peking University First Hospital, Beijing, China

**Keywords:** CSVD, distribution, MRI, connexins, gene polymorphism

## Abstract

Vascular endothelial cell (EC) and blood–brain barrier (BBB) dysfunction is the core pathogenesis of cerebral small vessel disease (CSVD). Moreover, animal experiments have shown the importance of connexin (Cx)-43 in EC and BBB function. In this study, we recruited 200 patients diagnosed with sporadic CSVD. Initially, we examined imaging scores of white matter hyperintensities (WMH), lacunar infarction (LI), and cerebral microbleeds (CMB). Additionally, we performed next-generation sequencing of the *GJA1* gene (Cx43 coding gene) to examine correlation between these single-nucleotide polymorphisms and the burden and distribution of CSVD. Fourteen target loci were chosen. Of these, 13 loci (92.9%) contributed toward risk for cerebellar LI, one locus (7.1%) was shown to be a protective factor for lobar CMB after FDR adjustment. In conclusion, single-nucleotide polymorphisms in the *GJA1* gene appear to affect the distribution but not severity of CSVD.

## Introduction

Cerebral small vessel disease (CSVD) affects the small arteries, arterioles, venules, and capillaries of the brain, and describes a group of clinical, pathological, and neuroimaging processes of heterogeneous etiologies (1). White matter hyperintensities (WMH), lacunar infarctions (LI), and cerebral microbleeds (CMB) are the three major neuroimaging features of CSVD (2). The topography of the cranial lesions (such as the incidence, severity, and location) differ depending on the disease origin. For example, in the most common inherited CSVD, cerebral autosomal dominant arteriopathy with subcortical infarcts and leukoencephalopathy (CADASIL), WMH tend to be located in the capsula externa and temporal pole (3). Contrarily, with age-related WMH, the lesions tend to involve the frontal lobes. These findings suggest that spatial specificity may provide insight into the underlying vascular pathology and disease mechanisms (4).

Dysfunction of vascular endothelial cells (ECs) and the blood–brain barrier (BBB), chronic ischemia/hypoperfusion and enlarged perivascular spaces play an important role in the pathogenesis of CSVD (5). Dysfunction of ECs may impact upon formation and repair of myelin (6), while breakdown of the BBB may lead to leakage of blood products, both of which can result in thickening and sclerosis of the arteriolar wall. The resultant loss of myelin and gliosis manifests as WMH on brain magnetic resonance imaging (MRI). Severe ischemia of the small vessel territory results in small subcortical infarctions. Leakage of blood products from a microaneurysm results in CMB (7). Therefore, the neuroimaging features of CSVD are closely related to its pathogenicity.

There are two types of vascular endothelial cell–cell junctions: the tight junction and adherens junction, with both playing key roles in the barrier function of the BBB (8, 9). Recently, researchers identified a third category, gap junction channels, that interact with other cell–cell junctions in maintenance of barrier function. Gap junctions appear as plaques at the cell plasma membrane surface and are formed by the interaction of two hemichannels. Each hemichannel is composed of six connexins (Cx), which are transmembrane proteins consisting of a cytoplasmic loop, two extracellular loops, a cytoplasmic C-terminal tail, and a cytoplasmic N-terminal tail. More than 20 different Cx species have been identified in humans. Cx43 is fully expressed across the central nervous system, especially in ECs, vascular smooth muscle cells, pericytes, and astrocytes, which are important components of the BBB (10). An increasing number of *in vivo* and *in vitro* experiments suggest that Cx43 is necessary for maintaining BBB function from several aspects including ECs (11, 12), astrocytes (13, 14), and hemichannels (15–17). Further, single-nucleotide polymorphisms (SNPs) of the *GJA1* gene (Cx43 coding gene) correlate with primary hypertension (locus rs1925223) (18), congenital heart disease (locus rs2071166) (19), and arrhythmia (locus rs1925223) (20). However, there is currently no clinical evidence showing a relationship between CSVD and Cx43, or any other Cx. In the present study, we performed next-generation sequencing of the *GJA1* gene to examine correlation between *GJA1* gene SNPs and brain MRI topography of WMH, LI, and CMB in CSVD. Our study provides clinical evidence for the role of Cx43 in the pathogenesis of CSVD.

## Materials and Methods

### Study Population

The Quanto 1.2 software will measure the sample size. Patients from the Neurology Department of Peking University First Hospital (Beijing, China) were recruited between 2013 and 2019. The inclusion criteria were: age ≥ 40 years-old; neuroimaging changes confirming CSVD, including at least one of the following changes—a recent small subcortical infarct, lacunae of presumed vascular origin, WMH of presumed vascular origin, enlarged perivascular space or CMB (2). Patients with large vessel stenosis [defined as stenosis > 50% detected by carotid ultrasound/transcranial doppler sonography (TCD) or magnetic resonance angiography (MRA) in any large or medium artery supplying brain tissue] or suspicion of cerebral amyloid angiopathy or Alzheimer's disease (based on clinical course or imaging changes) were excluded. Informed consent was obtained from all participants. This research was approved by the Ethics Committee of Peking University Health Science Center (IRB00001052-17018).

### Clinical Data

Demographic information of the patients (e.g., age, sex) was collected. Common vascular risk factors including hypertension, diabetes mellitus, dyslipidaemia, coronary heart disease, atrial fibrillation, renal function, serum homocysteine levels, smoking, family history of stroke, and use of antithrombotic drugs were documented. Color Doppler carotid ultrasound, TCD, and MRA were used to determine changes in extracranial and intracranial arteries. Venous blood was collected in EDTA anticoagulant tubes and stored at −80°C.

### Magnetic Resonance Scanning and Measuring

Each patient had undergone a head MR scan with sequences including T1-weighted, T2-weighted, fluid attenuated inversion recovery (FLAIR), diffusion weighted imaging (DWI), and T2^*^-weighted gradient-recalled echo (GRE). The MR scanners used were the General Electric Medical Systems 1.5 T (Chicago, IL, USA) and Philips Medical Systems 3.0 T (Amsterdam, the Netherlands). Axial slices at a slice thickness of 6.0 mm were obtained. One specifically trained rater analyzed the MRI data twice, then the second result was adopted. Key neuroimaging markers including WMH, LI (recent small subcortical infarcts and lacunae of presumed vascular origin), and CMB were examined, following the definition of STandards for ReportIng Vascular changes on nEuroimaging (STRIVE) (2). Perivascular spaces were not estimated in our study. The severity and distribution of WMH were assessed qualitatively and quantitatively. The age-related white matter changes (ARWMC) scale (21) was used to rate the severity and distribution of the lesions. The severity scale included mild (ARWMC scale < 7) and severe (ARWMC ≥ 7) (22). The number and location of LI were recorded. To count the number of lacunae, different areas were examined separately in the right and left hemispheres, that is, frontal, parietooccipital, temporal, corpus callosum, internal capsule, external capsule, basal ganglia, thalamus, brainstem, and cerebellum. The severity included single lacuna and multiple lacunae (number of LI ≥ 2). For microbleeds, the Microbleed Anatomical Rating Scale (MARS) (23) was used to record their number and distribution. The severity scale included mild (MARS < 5) and severe (MARS ≥ 5) (24).

### Next-Generation Sequencing and Functional Annotation

Genomic DNA was extracted from peripheral blood of all participants and next-generation sequencing of the *GJA1* gene was performed. DNA extraction, Ion Torrent Proton Library Preparation and Sequencing: Genomic DNA was extracted from anticoagulated whole blood of each sample using a commercial Blood Genomic DNA Miniprep kit (Axygen, Union City, CA, USA). The Qubit dsDNA HS Assay kit was used to quantify DNA with the QubitFluorometer (Thermo Fisher Scientific, Inc., Waltham, MA, USA), according to the manufacturer's instructions. 20 ng gDNA for each participant was amplified in two separate primer pool wells using Ion AmpliSeq Custom panel including 14.13kb Cx43 gene target region and AmpliSeq HiFi mix (Thermo Fisher Scientific,Inc, Carlsbad, CA, USA). All two PCR pools were combined in one well and subjected to primer digestion performing incubation with FuPa reagent (Thermo Fisher Scientific, Inc). Amplified Cx43 gene targets were ligated with Ion P1 and Ion Xpress Barcode adapters. After purification libraries were quantified using quantitative PCR with the Ion Library Quantification Kit (Thermo Fisher Scientific, Inc, Cat.no.446880). Sample emulsion PCR and enrichment were performed using the Ion PI HI-Q Template OT2 200 Kit (Thermo Fisher Scientific, Inc, Cat.no.A26434), according to the manufacturer's instructions. Template-positive ISPs were enriched and sequencing was undertaken using Ion PI HI-Q Sequencing 200 Kit (Termofisher, Cat.no.A26433) on the Ion Torrent Proton according to the manufacturer's instructions.

Tag loci were selected based on the allele frequency of variant nucleotides higher than the 1,000 Genomes Project East Asian allele frequency (25) and with a minor allele frequency (MAF) > 1% (26). Association of gene expression levels with genotype was further investigated using the online Genotype-Tissue Expression (GTEx) Portal expression quantitative trait locus (eQTL) browser (http://www.broadinstitute.org/gtex/). An eQTL represents a locus in the genome in which variation between individuals is correlated with a quantitative gene expression trait, often measured as mRNA abundance. The RegulomeDB database (https://regulomedb.org/regulome-search/) determines whether SNPs are located within known or predicted regulatory elements within intergenic regions of the human genome. Functional annotation data for SNPs were then obtained from HaploReg V4 (http://pubs.broadinstitute.org/mammals/haploreg/haploreg_v4.php).

### Statistical Analysis

Statistical analyses were performed using the Statistical Package for Social Sciences version 25.0 (IBM SPSS Statistics, Armonk, NY, USA). Depending on distribution of the values, quantitative data are expressed as mean ± standard deviation (SD) or median. Categorical data are described as frequencies and ratios. One-way analysis of variance (ANOVA) was used for comparison of age. Pearson's chi-squared test or Fisher's exact test were used to examine differences in categorical data. Multivariable regression analysis was performed to determine whether there is a significant difference after correcting for the effects of confounding factors such as age. Variables with *P* < 0.15 in univariable analyses and well-known risk factors of CSVD (age and hypertension) were included in multivariable analyses. *P-*values were controlled for multiple testing using FDR adjustment. *P* < 0.05 was considered significant.

## Results

### Baseline Information

Of 299 patients with sporadic CSVD, 93 patients were excluded: 4 patients for deficient vascular examinations, 62 patients for large vessel stenosis, 14 patients for cardiogenic embolism, 2 patients for demyelinating diseases, and 11 patients for rejecting participation. Overall, 206 patients conformed to the inclusion and exclusion criteria. However, a further 4 patients were excluded for data shortage and 2 patients for contaminated blood. Therefore, data of 200 patients were included in the final analysis (The power using Quanto for testing association using 200 samples is 0.81). Baseline information and neuroimaging features of the patients are shown in Supplementary Table 1. Altogether, 136 (68%) patients were male and the average age was 62.7 ± 11.0 years-old. Regarding disease status, 167 patients were diagnosed with hypertension, 64 (32.0%) with diabetes, 123 (61.5%) with dyslipidaemia, 28 (14.0%) with coronary heart disease, 61 (30.5%) with hyperhomocysteinemia, and 17 (8.5%) with chronic kidney disease. Moreover, 94 (47.0%) patients had a smoking history and 70 (35.0%) patients had a family history of cerebral vessel disease. Further, 67 (33.5%) patients were taking antiplatelet drugs, while 2 (1.0%) patients were taking anticoagulant drugs.

In total, 185 (92.5%) patients presented with WMH, with 97 (52.4%) patients exhibiting severe WMH. Of these WMH lesions, 177 (95.7%) were located in the frontal lobe, 143 (77.3%) in the parietooccipital lobe, 78 (42.2%) in the temporal lobe, 38 (20.5%) in the subtentorium, and 81 (43.8%) in the basal ganglia. Regarding LI, 157 (78.5%) patients presented with LI and 132 (84.1%) patients had multiple LI. Of the lacuna lesions, 103 (65.6%) were located in the frontal lobe, 32 (20.4%) in the parietooccipital lobe, 27 (17.2%) in the temporal lobe, 47 (29.9%) in the hippocampus, 125 (79.6%) in the basal ganglia, 8 (5.1%) in the cerebellum, and 39 (24.8%) in the brainstem. Regarding CMB, 138 patients had brain MRI performed with T2^*^-weighted GRE. Consequently, 45 (32.6%) patients had CMB, with severe CMB in 23 (51.1%). Of the CMB lesions, 28 (62.2%) were located in lobes, 33 (73.3%) in deep regions, and 21 (46.7%) in infratentorial regions.

### Target SNPs of the *GJA1* Gene

Overall, gene polymorphisms were detected in 44 loci. Subsequently, we selected 14 of them as target loci (Table 1).

**Table 1:** Target loci of the *GJA1* gene.

**Loci**	**Region**	***N* (variation point)**	**Allele frequency of variant nucleotide**	**TGP_EAS_AF**
rs3805787	intron	110	32.5%	30.4%
rs3805786	intron	91	26.3%	21.7%
rs3805785	intron	91	26.3%	21.4%
rs17083633	intron	91	26.3%	21.4%
rs3840372	intron	90	26.0%	21.4%
rs79597379	intron	90	25.8%	20.8%
rs76430488	intron	90	25.8%	21.4%
rs75464514	intron	90	25.8%	21.3%
rs149622654	intron	89	25.8%	20.8%
rs78125885	intron	89	25.6%	20.8%
rs3805788	intron	89	25.5%	20.6%
rs386705335	intron	88	25.3%	-
rs73532215	intron	87	25.3%	20.9%
rs72548744	UTR3	65	20.0%	15.0%

*TGP_EAS_AF, thousand-genome project_Easian_Allele frequency*.

### Hardy–Weinberg Equilibrium in *GJA1* Gene SNPs

For the 14 target loci, we calculated the genotype frequency of each case group and control group in WMH, LI, and CMB cohorts. We found no significance between the observed value and expected value for genotype distribution using the Hardy–Weinberg equilibrium test. Therefore, all SNPs of target loci can be assumed to represent the population.

### Correlation Between SNPs and CSVD

#### Correlation Between SNPs and WMH

The patients were classified into three groups based upon Cx43 genotype, namely, wild-type homozygous genotype, variant homozygous genotype, and variant heterozygous genotype. We then compared the severity and distribution of WMH among the three groups. There were no significant differences among the three genotypes in the severity or spatial distribution of all 14 loci. We then set the risk factors [*P* < 0.15 in univariable analyses and well-known risk factors of CSVD (age and hypertension)] as confounding factors (shown in Table 2). After adjusting for confounding factors (multivariable analysis shown in Table 2), we found that WMH tended to be located in the temporal lobe with the variant homozygous genotype compared with the wild-type homozygous genotype for loci rs78125885, rs76430488/rs75464514 (genotype distribution was the same), rs3805788, rs386705335, rs72548744, and rs79597379. Moreover, for locus rs73532215, WMH tended to be located in the infratentorial region for the variant homozygous genotype compared with the wild-type homozygous genotype. There were no significant differences in the severity of WMH among the three genotypes. However, after FDR adjustment, SNPs of all 14 loci were not associated with distribution or severity of WMH (*P* > 0.05).

**Table 2:** Association of *GJA1* gene SNPs and white matter hyperintensities (multivariable analysis).

**Loci**	**Severe WMH**			**Frontal lobe**			**Parietooccipital lobe**			**Temporal lobe**			**Infratentorial region**			**Basal ganglia**		
	**OR^**a**^,95%CI**	***P***	P^*^	**OR^**a**^,95%CI**	***P***	P^*^	**OR^**a**^,95%CI**	***P***	P^*^	**OR^**a**^,95%CI**	***P***	P^*^	**OR^**a**^,95%CI**	***P***	P^*^	**OR^**a**^,95%CI**	***P***	P^*^
**rs78125885**^**c**^																		
AA(*N* = 111)	1.00	-		1.00	-		1.00	-		1.00	-		1.00	-		1.00	-	
AG(*N* = 76)^b^	0.81(0.43–1.53)	0.52	0.73	0.96(0.21–4.35)	0.52	1.00	0.73(0.34–1.56)	0.41	0.64	1.19(0.62–2.27)	0.61	0.94	1.10(0.49–2.46)	0.82	0.98	1.41(0.75–2.67)	0.29	0.70
GG(*N* = 13)^b^	1.80(0.23–2.76)	0.73	1.00	-	1.00	1.00	0.63(0.15–2.69)	0.53	0.82	4.18(1.09–16.00)	0.04	0.08	2.63(0.66–10.53)	0.17	0.37	1.69(0.50–5.64)	0.40	0.97
**rs76430488/rs75464514**^**d**^																		
TT(*N* = 110)	1.00	-		1.00	-		1.00	-		1.00	-		1.00	-		1.00	-	
TC(*N* = 77)^b^	0.72(0.38–1.36)	0.31	1.00	0.54(0.12–2.31)	0.40	1.00	0.71(0.33–1.51)	0.37	0.64	1.05(0.55–2.01)	0.89	0.94	1.09(0.49–2.45)	0.84	0.98	1.26(0.67–2.38)	0.47	0.70
CC(*N* = 13)^b^	0.79(0.23–2.74)	0.71	1.00	-	1.00	1.00	0.67(0.15–2.88)	0.58	0.82	4.01(1.04–15.50)	0.04	0.08	3.05(0.73–12.75)	0.13	0.37	1.68(0.50–5.68)	0.40	0.97
**rs3805788**^**e**^																		
CC(*N* = 111)	1.00	-		1.00	-		1.00	-		1.00	-		1.00	-		1.00	-	
CT(*N* = 76)^b^	0.77(0.41–1.45)	0.42	1.00	0.92(0.20–4.17)	0.91	1.00	0.81(0.38–1.75)	0.60	0.69	1.10(0.57–2.12)	0.77	0.94	0.96(0.43–2.18)	0.93	0.98	1.33(0.71–2.52)	0.38	0.70
TT(*N* = 13)^b^	0.78(0.23–2.69)	0.70	1.00	-	1.00	1.00	0.66(0.16–2.82)	0.58	0.82	4.05(1.06–15.52)	0.04	0.08	2.48(0.62–9.89)	0.20	0.37	1.64(0.49–5.50)	0.42	0.97
**rs73532215**^**f**^																		
AA(*N* = 113)	1.00	-		1.00	-		1.00	-		1.00	-		1.00	-		1.00	-	
AG(*N* = 73)^b^	0.84(0.45–1.60)	0.60	0.65	0.92(0.20–4.20)	0.92	1.00	0.76(0.35–1.63)	0.48	0.67	1.22(0.63–2.36)	0.56	0.94	0.75(0.32–1.76)	0.51	0.98	1.32(0.69–2.52)	0.39	0.70
GG(*N* = 14)^b^	0.90(0.27–3.02)	0.87	1.00	-	1.00	1.00	0.65(0.15–2.76)	0.56	0.82	3.10(0.88–10.87)	0.08	0.09	4.14(1.04–16.47)	0.04	0.37	1.82(0.55–5.98)	0.33	0.97
**rs386705335**^**g**^																		
GCGC(*N* = 112)	1.00	-		1.00	-		1.00	-		1.00	-		1.00	-		1.00	-	
GCAT(*N* = 75)^b^	0.82(0.43–1.54)	0.53	0.67	0.89(0.20–4.04)	0.88	1.00	0.79(0.37–1.69)	0.54	0.69	1.16(0.60–2.24)	0.67	0.94	1.01(0.44–2.29)	0.98	0.98	1.40(0.74–2.66)	0.30	0.70
ATAT(*N* = 13)^b^	0.80(0.23–2.77)	0.73	1.00	-	1.00	1.00	0.65(0.15–2.78)	0.56	0.82	4.14(1.08–15.87)	0.04	0.08	2.53(0.63–10.11)	0.19	0.37	1.67(0.50–5.61)	0.40	0.97
**rs72548744**^**h**^																		
GG(*N* = 135)	1.00	-		1.00	-		1.00	-		1.00	-		1.00	-		1.00	-	
GA(*N* = 50)^b^	0.57(0.28–1.17)	0.13	1.00	-	1.00	1.00	0.82(0.36–1.91)	0.66	0.69	0.91(0.44–1.90)	0.80	0.94	1.47(0.61–3.53)	0.39	0.91	1.15(0.57–2.33)	0.70	0.70
AA(*N* = 15)^b^	0.68(0.23–2.03)	0.49	1.00	0.67(0.08–6.02)	0.72	1.00	1.10(0.28–4.36)	0.89	0.89	3.49(1.05–11.58)	0.04	0.08	2.90(0.84–9.98)	0.09	0.37	1.16(0.39–3.49)	0.79	0.97
**rs79597379**^**i**^																		
CC(*N* = 110)	1.00	-		1.00	-		1.00	-		1.00	-		1.00	-		1.00	-	
CT(*N* = 77)^b^	0.70(0.38–1.32)	0.27	1.00	0.73(0.28–1.89)	0.52	1.00	0.72(0.35–1.48)	0.37	0.64	1.42(0.73–2.78)	0.31	0.72	1.04(0.44–2.45)	0.93	0.98	0.57(0.30–1.08)	0.09	0.63
TT(*N* = 13)^b^	1.45(0.38–5.49)	0.58	1.00	-	1.00	1.00	0.68(0.15–3.09)	0.62	0.82	5.30(1.28–21.9)	0.02	0.08	2.24(0.48–10.47)	0.31	0.37	0.98(0.33–2.88)	0.97	0.97

a *Logistic regression analysis, P < 0.05 was considered significant*.

b *Compared with wild-type homozygous genotype*.

* *P-value after FDR adjustment*.

c,d,e,g,i The confounding factors were age, hypertension, dyslipidaemia, chronic kidney disease, and use of anticoagulant drugs;

f age, hypertension, dyslipidaemia, smoking history, chronic kidney disease, and use of anticoagulant drugs;

h *the confounding factor was chronic kidney disease*.

*CI, confidence interval*.

#### Correlation Between SNPs and LI

Again, the patients were distributed into three groups based upon Cx43 genotype, and the severity and distribution of LI compared among the three groups. Before adjusting for confounding factors, we detected significant differences in cerebellar distribution of LI with loci rs79597379, rs72548744, rs34782269, rs76430488/rs75464514 (genotype distribution was the same), rs78125885, rs73532215, rs386705335, rs3840372, rs3805786/rs3805785/rs17083633 (genotype distribution was the same), rs3805788, and rs14962265 (*P* < 0.05). Further, there were significant differences in parietooccipital lobe distribution of LI with loci rs72548744 and rs73532215 (*P* < 0.05). There were no significant differences in the severity of LI. Positive results are shown in Table 3.

**Table 3:** *GJA1* gene SNPs and lacunar infarctions.

**MRI features**	**Loci**			
**rs79597379**				
	CC(*N* = 110)	CT(*N* = 77)	TT(*N* = 13)	*P*
Cerebellum	3(3.7%)	2(3.1%)	3(27.3%)	0.02
**rs72548744**				
	GG(*N* = 135)	GA(*N* = 50)	AA (*N* = 15)	*P*
Parietooccipital lobe	20(19.2%)	6(14.6%)	6(50.0%)	0.04
Cerebellum	4(3.8%)	1(2.4%)	3(25.0%)	0.03
**rs76430488/ rs75464514**				
	TT(*N* = 110)	TC(*N* = 77)	CC(*N* = 13)	*P*
Cerebellum	3(3.7%)	2(3.1%)	3(27.3%)	0.02
**rs78125885**				
	AA(*N* = 111)	AG(*N* = 76)	GG(*N* = 13)	*P*
Cerebellum	3(3.7%)	2(3.1%)	3(27.3%)	0.02
**rs73532215**				
	AA(*N* = 113)	AG(*N* = 73)	GG(*N* = 14)	*P*
Cerebellum	3(3.6%)	2(3.3%)	3(25.0%)	0.03
Parietooccipital lobe	18(21.4%)	8(13.1%)	5(50.0%)	0.02
**rs386705335**				
	GCGC(*N* = 112)	GCAT(*N* = 75)	ATAT(*N* = 13)	*P*
Cerebellum	3(3.6%)	2(3.2%)	3(27.3%)	0.02
**rs3840372**				
	–(110)	-T(76)	TT(14)	*P*
Cerebellum	3(3.7%)	2(3.1%)	3(27.3%)	0.02
**rs3805786/rs3805785/rs17083633**				
	AA(109)	AC(77)	CC(14)	*P*
Cerebellum	3(3.7%)	2(3.1%)	3(27.3%)	0.02
**rs3805788**				
	CC(111)	CT(76)	TT(13)	*P*
Cerebellum	3(3.6%)	2(3.2%)	3(27.3%)	0.02

*Pearson's chi-squared test or Fisher's exact test were used. P < 0.05 was considered significant*.

*MRI, magnetic resonance imaging*.

After adjusting for confounding factors (multivariable analysis shown in Table 4). Moreover, LI tended to be cerebellar in location with the variant homozygous genotype compared with wild-type homozygous genotype at loci rs79597379, rs72548744, rs76430488/rs75464514, rs78125885, rs73532215, rs386705335, rs3840372, rs3805786/rs3805785/rs17083633, rs3805788, and rs149622654. Meanwhile, for loci rs72548744 and rs73532215, LI tended to be located in the parietooccipital lobe with the variant homozygous genotype compared with the wild-type homozygous genotype. There were no significant differences in severity of LI among the three Cx43 genotypes. After FDR adjustment, SNPs of locus rs79597379, rs72548744, rs76430488/rs75464514, rs78125885, rs386705335, rs3840372, rs3805786/rs3805785/rs17083633, rs3805788, and rs149622654 (*P* = 0.023) and rs73532215 (*P* = 0.043) was still associated with cerebellar LI.

**Table 4:** Association of *GJA1* gene SNPs and distribution of lacunar infarctions (multivariable analysis).

**Loci**	**Frontal lobe**			**Parietooccipital lobe**			**Temporal lobe**			**Hippocampus**			**Basal ganglia**			**Cerebellum**			**Brain stem**		
	**OR^**a**^,95%CI**	**P**	P^*^	**OR^**a**^,95%CI**	**P**	P^*^	**OR^**a**^,95%CI**	**P**	P^*^	**OR^**a**^,95%CI**	**P**	P^*^	**OR^**a**^,95%CI**	**P**	P^*^	**OR^**a**^,95%CI**	**P**	P^*^	**OR^**a**^,95%CI**	**P**	P^*^
**rs79597379**^**c**^																					
CC(*N* = 110)	1.00	-		1.00	-		1.00	-		1.00	-		1.00	-		1.00	-		1.00	-	
CT(*N* = 77)^b^	0.94(0.46–1.90)	0.86	1.00	0.77(0.32–1.84)	0.55	0.71	0.71(0.29–1.78)	0.47	0.78	1.09(0.53–2.27)	0.81	0.87	0.55(0.24–1.25)	0.41	1.00	0.77(0.12–4.96)	0.79	0.93	1.22(0.56–2.67)	0.63	1.00
TT(*N* = 13)^b^	0.99(0.23–4.31)	0.98	1.00	3.28(0.82–13.08)	0.09	0.10	1.16(0.21–6.28)	0.87	0.97	1.68(0.42–6.73)	0.46	0.58	-	1.00	1.00	9.48(1.43–62.98)	0.02	***0.023***	1.11(0.24–5.13)	0.90	1.00
**rs72548744**^**d**^																					
GG(*N* = 135)	1.00	-		1.00	-		1.00	-		1.00	-		1.00	-		1.00	-		1.00	-	
GA(*N* = 50)^b^	1.48(0.66–3.31)	0.35	1.00	0.77(0.28–2.12)	0.61	0.71	0.56(0.19–1.67)	0.30	0.78	1.90(0.87–4.14)	0.11	0.87	0.94(0.37–2.39)	0.90	0.90	0.63(0.07–6.14)	0.69	0.93	0.58(0.23–1.45)	0.24	1.00
AA(*N* = 15)^b^	1.67(0.42–6.66)	0.47	1.00	4.15(1.20–14.36)	0.03	0.10	0.85(0.17–4.26)	0.85	0.97	0.54(0.11–2.65)	0.45	0.58	1.48(0.29–7.51)	0.64	1.00	7.31(1.34–40.00)	0.02	***0.023***	0.23(0.03–1.88)	0.17	1.00
**rs76430488 / rs75464514**^**e**^																					
TT(*N* = 110)	1.00	-		1.00	-		1.00	-		1.00	-		1.00	-		1.00	-		1.00	-	
TC(*N* = 77)^b^	0.89(0.44–1.78)	0.74	1.00	0.75(0.31–1.79)	0.52	0.71	0.79(0.33–1.92)	0.61	0.78	1.13(0.55–2.32)	0.75	0.87	0.68(0.30–1.52)	0.35	0.41	0.77(0.12–4.95)	0.78	0.93	1.03(0.48–2.25)	0.93	1.00
CC(*N* = 13)^b^	1.08(0.25–4.61)	0.92	1.00	3.40(0.86–13.46)	0.08	0.10	1.05(0.20–5.61)	0.96	0.97	1.69(0.40–6.38)	0.50	0.58	-	1.00	1.00	9.48(1.43–62.98)	0.02	***0.023***	1.03(0.23–4.67)	0.97	1.00
**rs78125885**^**f**^																					
AA(*N* = 111)	1.00	-		1.00	-		1.00	-		1.00	-		1.00	-		1.00	-		1.00	-	
AG(*N* = 76)^b^	0.94(0.46–1.90)	0.86	1.00	0.77(0.32–1.84)	0.55	0.71	0.71(0.29–1.78)	0.47	0.78	1.09(0.53–2.27)	0.81	0.87	0.55(0.24–1.25)	0.15	0.41	0.77(0.12–4.96)	0.79	0.93	1.22(0.56–2.67)	0.63	1.00
GG(*N* = 13)^b^	0.99(0.23–4.31)	0.98	1.00	3.28(0.82–13.08)	0.09	0.11	1.16(0.21–6.28)	0.87	0.97	1.68(0.42–6.73)	0.46	0.58	-	1.00	1.00	9.48(1.43–62.98)	0.02	***0.023***	1.11(0.24–5.13)	0.90	1.00
**rs73532215**^**g**^																					
AA(*N* = 113)	1.00	-		1.00	-		1.00	-		1.00	-		1.00	-		1.00	-		1.00	-	
AG(*N* = 73)^b^	0.91(0.45–1.83)	0.79	1.00	0.58(0.23–1.47)	0.25	0.71	0.89(0.36–2.17)	0.79	0.85	1.21(0.58–2.52)	0.61	0.87	0.62(0.28–1.40)	0.25	0.41	0.93(0.14–5.98)	0.94	0.94	1.22(0.56–2.68)	0.62	1.00
GG(*N* = 14)^b^	1.24(0.30–5.17)	0.77	1.00	3.95(1.03–15.14)	0.04	0.10	0.89(0.17–4.73)	0.89	0.97	1.53(0.39–5.99)	0.54	0.58	-	1.00	1.00	6.74(1.04–43.63)	0.04	***0.043***	0.94(0.21–4.19)	0.93	1.00
**rs386705335**^**h**^																					
GCGC(*N* = 112)	1.00	-		1.00	-		1.00	-		1.00	-		1.00	-		1.00	-		1.00	-	
GCAT(*N* = 75)^b^	0.96(0.48–1.93)	0.91	1.00	1.12(0.26–4.78)	0.88	0.88	0.82(0.34–1.99)	0.66	0.78	1.17(0.57–2.42)	0.67	0.87	0.65(0.29–1.46)	0.30	0.41	0.78(0.12–5.05)	0.80	0.93	1.08(0.49–2.35)	0.85	1.00
ATAT(*N* = 13)^b^	0.78(0.32–1.86)	0.57	1.00	3.46(0.87–13.70)	0.08	0.10	1.06(0.20–5.70)	0.94	0.97	1.64(0.41–6.50)	0.49	0.58	-	1.00	1.00	9.55(1.44–63.43)	0.02	***0.023***	1.05(0.23–4.76)	0.95	1.00
**rs3840372**^**i**^																					
(*N* = 110)	1.00	-		1.00	-		1.00	-		1.00	-		1.00	-		1.00	-		1.00	-	
T(*N* = 76)^b^	0.93(0.47–1.85)	0.83	1.00	0.73(0.31–1.74)	0.48	0.71	0.82(0.34–1.99)	0.67	0.78	1.13(0.55–2.33)	0.73	0.87	0.71(0.32–1.56)	0.39	0.41	0.84(0.13–5.30)	0.86	0.93	1.00(0.46–2.16)	1.00	1.00
TT(*N* = 14)^b^	1.18(0.28–4.98)	0.82	1.00	3.24(0.84–12.46)	0.09	0.10	1.14(0.22–5.98)	0.88	0.97	1.63(0.42–6.39)	0.48	0.58	-	1.00	1.00	10.38(1.58–68.41)	0.02	***0.023***	0.91(0.20–4.07)	0.90	1.00
**rs3805786/rs3805785/rs17083633**^**j**^																					
AA(*N* = 109)	1.00	-		1.00	-		1.00	-		1.00	-		1.00	-		1.00	-		1.00	-	
AC(*N* = 77)^b^	0.89(0.44–1.78)	0.74	1.00	0.75(0.31–1.79)	0.52	0.71	0.79(0.33–1.92)	0.61	0.78	1.13(0.55–2.32)	0.75	0.87	0.68(0.30–1.52)	0.35	0.41	0.77(0.12–4.95)	0.78	0.93	1.03(0.48–2.25)	0.93	1.00
CC(*N* = 14)^b^	1.08(0.25–4.61)	0.92	1.00	3.40(0.86–13.46)	0.08	0.10	1.05(0.20–5.61)	0.96	0.97	1.60(0.40–6.38)	0.50	0.58	-	1.00	1.00	9.48(1.43–62.98)	0.02	***0.023***	1.03(0.23–4.67)	0.97	1.00
**rs3805788**^**k**^																					
CC(*N* = 111)	1.00	-		1.00	-		1.00	-		1.00	-		1.00	-		1.00	-		1.00	-	
CT(*N* = 76)^b^	0.96(0.48–1.93)	0.91	1.00	0.78(0.32–1.86)	0.57	0.71	0.66(0.27–1.64)	0.37	0.78	1.02(0.49–2.11)	0.95	0.95	0.65(0.20–1.46)	0.30	0.41	0.78(0.12–5.04)	0.80	0.93	1.08(0.49–2.35)	0.85	1.00
TT(*N* = 13)^b^	1.12(0.26–4.78)	0.88	1.00	3.46(0.87–13.70)	0.08	0.10	0.97(0.18–5.19)	0.97	0.97	1.53(0.39–6.07)	0.55	0.58	-	1.00	1.00	9.54(1.44–63.43)	0.02	***0.023***	1.05(0.23–4.76)	0.95	1.00
**rs149622654**^**l**^																					
TTTT(*N* = 111)	1.00	-		1.00	-		1.00	-		1.00	-		1.00	-		1.00	-		1.00	-	
TT–(*N* = 76)^b^	0.96(0.48–1.93)	0.91	1.00	0.78(0.32–1.86)	0.57	0.71	0.82(0.34–1.99)	0.66	0.78	1.17(0.57–2.42)	0.67	0.87	0.65(0.29–1.46)	0.30	0.41	0.78(0.12–5.05)	0.80	0.93	1.08(0.49–2.35)	0.85	1.00
(*N* = 15)^b^	1.12(0.26–4.78)	0.88	1.00	3.46(0.87–13.70)	0.08	0.10	1.06(0.20–5.70)	0.94	0.97	1.64(0.41–6.50)	0.49	0.58	-	1.00	1.00	9.55(1.44–63.43)	0.02	***0.023***	1.05(0.23–4.76)	0.95	1.00

a *Logistic regression analysis, P < 0.05 was considered significant*.

b *Compared with wild-type homozygous genotype*.

* *P-value after FDR adjustment*.

c,e,f,j,k,l the confounding factors were age, hypertension, dyslipidaemia, chronic kidney disease, and use of anticoagulant drugs;

d chronic kidney disease; age, hypertension, dyslipidaemia, smoking history, chronic kidney disease, and use of anticoagulant drugs;

h,i *age, hypertension, chronic kidney disease, and use of anticoagulant drugs*.

*CI, confidence interval*.

#### Correlation Between SNPs and CMB

Overall, 138 patients underwent brain MRI with T2^*^-weighted GRE. Similarly, the patients were classified into three groups based upon Cx43 genotype, and the severity and distribution of CMB compared among the three groups. Before adjusting for confounding factors, there were significant differences in lobar region distribution of CMB with loci rs76430488/rs75464514 and rs73532215 (*P* < 0.05). However, there were no significant differences in the severity of CMB (*P* > 0.05). Positive results are shown in Table 5.

**Table 5:** *GJA1* gene SNPs and cerebral microbleeds.

**MRI features**	**Loci**			
**rs76430488/ rs75464514**				
Lobar region	TT(*N* = 76)	TC(*N* = 51)	CC(*N* = 11)
	15(65.2%)	12(80.0%)	1(14.3%)	0.01
**rs73532215**				
	AA(*N* = 113)	AG(*N* = 73)	GG(*N* = 14)	*p*
Lobar region	15(65.2%)	12(80.0%)	1(14.3%)	0.01

*Pearson's chi-squared test or Fisher's exact test were used. P < 0.05 was considered significant*.

*MRI, magnetic resonance imaging*.

After adjusting for confounding factors (multivariable analysis shown in Table 6), we found that CMB tended not to be located in the lobar region with the variant homozygous genotype compared with wild-type homozygous genotype at locus rs73532215 (*P* = 0.03, OR = 0.06, 95%CI, 0.01–0.75). There were no significant differences in the severity of CMB among the three genotypes. After FDR adjustment, SNP of locus 73532215 was still negative associated with lobar CMB (*P* = 0.04).

**Table 6:** Association of *GJA1* gene SNPs and cerebral microbleeds (multivariable analysis).

**Loci**	**Severity**			**Lobar region**			**Deep region**			**Infratentorial region**		
	**OR^**a**^,95%CI**	***P***	P^*^	**OR^**a**^,95%CI**	***P***	P^*^	**OR^**a**^,95%CI**	***P***	P^*^	**OR^**a**^,95%CI**	***P***	P^*^
**rs76430488/rs75464514**^**c**^												
TT(*N* = 76)	1.00	-		1.00	-		1.00	-		1.00	-	
TC(*N* = 51)^b^	1.28(0.33–5.00)	0.72	0.72	3.33(0.57–19.46)	0.18	0.21	0.61(0.14–2.63)	0.50	1.00	1.16(0.30–4.54)	0.83	0.83
CC(*N* = 11)^b^	0.30(0.04–2.15)	0.23	0.29	0.09(0.01–1.08)	0.06	0.07	1.53(0.14–16.96)	0.73	0.85	0.34(0.05–2.52)	0.29	0.37
**rs73532215**^**d**^												
AA(*N* = 78)	1.00	-		1.00	-		1.00	-		1.00	-	
AG(*N* = 47)^b^	1.32(0.33–5.25)	0.70	0.72	4.72(0.73–30.79)	0.11	0.15	0.49(0.10–2.34)	0.37	1.00	1.17(0.30–4.67)	0.82	0.83
GG(*N* = 13)^b^	0.29(0.04–2.13)	0.22	0.29	*0.06(0.01–0.75)*	*0.03*	***0.04***	1.88(0.15–22.93)	0.62	0.85	0.34(0.05–2.54)	0.29	0.37

a *Logistic regression analysis, P < 0.05 was considered significant*.

b *Compared with wild-type homozygous genotype*.

* *P-value after FDR adjustment*.

c the confounding factors were age, hypertension, dyslipidaemia, chronic kidney disease, and use of anticoagulant drugs;

d *age, hypertension, dyslipidaemia, smoking history, chronic kidney disease, and use of anticoagulant drugs*.

*CI, confidence interval*.

Thus, in summary, homozygosis of 13 (13/14, 92.9%) loci in the *GJA1* gene were probably the risk factor for cerebellar LI. Homozygosis of one (1/14, 7.1%) loci was probably the protective factor of lobar CMB. There were SNPs of each loci that did not correlate with severity of WMH, LI, or CMB.

### Functional Annotation of *GJA1* Gene SNPs

In the GTEx database, there were no significant differences between Cx43 genotype and gene expression in brain tissue (*P* > 0.05) with very small size of 20 heterozygosis variation and 0 or 1 homozygosis variation. This suggests that gene expression of the *GJA1* gene in brain tissue does not correlate with genotype. No evidence was detected for expression in cerebral vessels.

Data in the HaploReg and Regulome database are shown in Table 7. Annotation in HaploReg showed that SNPs of all 14 loci might influence gene expression of the *GJA1* gene. Overall, 12 loci overlapped the promotor modifying zone of histone methylation and acetylation, 13 loci overlapped the enhancer modifying zone of histone, and 10 loci overlapped hypersensitive DNase sites (which can be cut by DNase, suggesting they are located in transcription factor active zones). By restricting the tissue to the brain or cerebral vessels, we still identified 11 loci overlapping the promotor modifying zone of histone methylation and acetylation, 10 loci overlapping the enhancer modifying zone of histone, and 3 loci overlapping hypersensitive DNase sites. Five loci were predicted to bind protein and 11 loci could cause motif changes. Predicting outcomes of RegulomeDB showed that loci rs3805786 and rs3805788 may influence the binding of transcription factors.

**Table 7:** Prediction of functional variation in HaploReg and RegulomeDB.

**SNPs**	**HaploReg**					**Regolume DB score^**a**^**
	**Promotor modify^**a**^**	**Enhancer modify^**a**^**	**Hypersensitive site of DNAse**^**a**^	**Protein binding**^**b**^	**Changes of motifs**^**c**^	
rs79597379	20, +	2	3		5	5
rs78125885	20, +	2	2	-	-	4
rs72548744	-	2	-	-	3	6
rs76430488	10, +	4, +	2	-	1	5
rs75464514	1	5, +	-	-	17	5
rs73532215	22, +	1, +	35, +	4	-	4
rs386705335	22, +	1, +	22, +	4	4	4
rs3840372	15, +	3, +	1	-	6	-
rs3805785	7, +	12, +	-	-	9	6
rs3805786	13, +	7, +	4	-	18	2b
rs17083633	10,+	11, +	2	3	1	-
rs3805788	21,+	2,+	2	3	11	2b
rs149622654	20,+	1,+	22, +	4	4	4

a *Representing the number of cell lines in different tissues; + shows the cell line including brain and cerebral tissue*.

b *Number of protein binding sites*.

c *Changes of predicted motifs*.

d *Regulome DB: 1–6, higher score indicates less evidence influencing transcription factor binding. 2b indicates a probable binding influence*.

*SNPs, single nucleotide polymorphism*.

## Discussion

Our study shows that SNPs of Cx43 correlate with the burden of CSVD. We focused on the impact of Cx43 in CSVD mainly because Cx43 is highly expressed in vascular ECs and ECs in the BBB, as well as astrocytes and pericytes forming the BBB (10). *GJA4* (Cx37 coding gene) gene SNPs correlate with large vessel disease, such as ischemic stroke and coronary heart disease (27, 28). Similarly, *GJA1* gene SNPs correlate with primary hypertension, congenital heart disease, and arrhythmia (18–20). Although there is no clear clinical evidence suggesting that *GJA1* gene SNPs correlate with CSVD, its biodistribution supports this hypothesis. In the present study, we show that *GJA1* gene SNPs mainly influence the distribution of LI and CMB lesions, with less effect on WMH.

*GJA1* gene SNPs mostly influenced lesion distribution and there was no correlation with lesion severity. First, heterogeneity of spatial distribution may be caused by uneven BBB regulation in different brain areas (29). Using a mouse model, Villasenor found region-specific differences in permeability upon loss of pericytes, with marked increases in permeability in the cortex, hippocampus, and corpus striatum. This might be caused by region-specific intracellular information delivery (30). Therefore, the influence of Cx43 on localization of CSVD is probably because of differing regulation of information delivery leading to altered permeability in different brain regions. Second, specific spatial distribution might be caused by differences in anatomical structures. Zhao et al. found differing densities of blood capillaries in different brain regions, being highest in quadrigeminal bodies, followed by the thalamus, parietooccipital cortex, frontal cortex, hippocampus, corpus striatum, midbrain, and cerebellum. Densities in the pons and medulla were lowest (31). This suggests that region-specific permeability of the BBB might correlate with different expression of Cx. Third, different structures exhibit different susceptibility to BBB dysfunction. Using an osmotic demyelination syndrome model of mice, Bouchat et al. found that central nervous system demyelination might be caused by regional vulnerability of oligodendrocytes and astrocytes, and refuted BBB disruption as a primary cause of demyelination, especially in the thalamus and occipital cortex (32). Consequently, *GJA1* gene SNPs might influence localization of CSVD via differing expression of Cx43 and differential regulation of information delivery in different brain regions. Our findings still need further experiments to investigate the expression of Cx43 mRNA and protein in the cerebellar region.

Cx43 might differentially influence multiple pathways of CSVD. Our research suggests that *GJA1* gene SNPs mostly affect LI, followed by CMB. These opposing actions might be caused by different pathogenic pathways in various types of CSVD. Dysfunction of ECs leads to stenosis of cerebral vessels and acute ischemia, further promoting the likelihood of LI. Breakdown of the BBB causes leakage of plasma substances, while chronic hypoperfusion inhibits the generation and repair of myelin, thereby injuring the axon and causing WMH. CMB are caused by BBB breakdown or amyloid β (Aβ) deposition during cerebral amyloid angiopathy (CAA) (3, 33). Our findings suggest that *GJA1* gene SNPs are mainly associated with EC pathways or Aβ deposition.

Our results suggest a key role of Cx43 in development of cranial lesions in CSVD. We also provide clinical evidence to support a critical function of Cx43 on ECs. Li et al. found that downregulation of Cx43 inhibited differentiation of endothelial progenitor cells and neointima formation, and ultimately inhibited vessel repair (11). Meanwhile, another study found that inhibition of Cx43 activity inhibited proliferation and migration by inhibiting extracellular signal-regulated kinase 1/2 (12). Further, Chen et al. showed that Cx43/Zo1 complex (specifically, a tight junction) maintained the balance of directional migration control to a more linear movement, which enhanced the rate of wound healing (34). In cell experiments, Saez et al. found that reduction of hemichannel activity may represent a strategy against the activation of deleterious pathways leading to endothelial dysfunction (16). When Cx43 activities are disrupted, regulation of EC function is lost, and LI occurs. Cx43 can also affect BBB function by both protein expression and opening of hemichannels. Ezan et al. found that decreased Cx43 expression weakened BBB function (13). Corroboratively, another two studies have shown that opening of Cx43 hemichannels can cause dysfunction of information delivery in the BBB by leakage of intracellular substances and ATP (17, 35). Subsequently, impairment of BBB permeability leads to WMH and CMB. Unfortunately, in our study, there is no correlation between *GJA1* gene and WMH. It is worth noting that, the GG genotype of rs73532215 is negative associated with CMB. Yi et al. found that connexin43 gene knocking-out mice allowed to reduce oxidative stress and neuritic dystrophies in hippocampal neurons associated to Aβ plaques (36). This suggested that GG genotype of rs73532215 might reduce the impairment of vessels caused by Aβ deposition to lower the risk of lobar CMB. Large sample researches are needed.

In GTEx database, we found no significant differences between Cx43 genotype and gene expression in brain tissue. However, the sample size in GTEx database was small, the significance was limited. In HaploReg database, we found that 13 SNPs were proximal to known regulatory regions that might influence gene expression of the *GJA1* gene. Large sample size and further experiments *in vivo* and *vitro* are needed.

## Conclusion

In summary, we have shown that *GJA1* gene SNPs correlate with MRI features of CSVD. *GJA1* gene SNPs mainly influence the distribution of LI in cerebellum, a single locus might be a protective factor for lobar CMB. Thus, the *GJA1* gene might regulate the distribution of CSVD to some extent. Our study provides the first clinical evidence of an association between *GJA1* gene SNPs and CSVD. Based on previous experiments, we further show that Cx43 correlates with clinical destruction of EC function. Moreover, we investigated region-specific differences among different genotypes of *GJA1* gene SNPs and show the impact of Cx43 on spatial heterogeneity of CSVD.

## Data Availability Statement

The original contributions presented in the study are publicly available. This data can be found at: www.ncbi.nlm.nih.gov, PRJNA672247.

## Ethics Statement

The studies involving human participants were reviewed and approved by the Ethics Committee of Peking University (PU IRB), No. IRB00001052-17018. The patients/participants provided their written informed consent to participate in this study. Written informed consent was obtained from the individual(s) for the publication of any potentially identifiable images or data included in this article.

## Author Contributions

JZ participated in data collection, performed the imaging evaluation and statistical analysis, interpreted the results, and drafted the manuscript. QY and WS participated in data collection in Beijing. JS and HJ contributed to the statistical analysis. QG and MY provided guidance and advice for genetic analysis. JS, WZ, and YH were involved in design of the study, supervised the data collection process, analysis, and interpretation of data, and revised the manuscript for intellectual content. All authors contributed to the article and approved the submitted version.

## Conflict of Interest

The authors declare that the research was conducted in the absence of any commercial or financial relationships that could be construed as a potential conflict of interest.
