# Supplementary material for: GJA1 Gene Polymorphisms and Topographic Distribution of Cranial MRI Lesions in Cerebral Small Vessel Disease
Source: Front Neurol. 2020 Nov 25;11:583974. doi: 10.3389/fneur.2020.583974 (PMC7723976; doi:10.3389/fneur.2020.583974)
Supplement: Supplementary file 1 [file Table_1.docx]

**Connexin 43 gene polymorphisms and topographic distribution of cranial MRI lesions in cerebral small vessel disease**

Jing Zhang, Qian You, Junlong Shu, Haiqiang Jin, Qiang Gang, Meng Yu, Wei Sun, Wei Zhang, and Yining Huang

**Supplementary Table S1 Baseline information and neuroimaging features of patients with cerebral small vessel disease**

|  | | | | N (%) | |
| --- | --- | --- | --- | --- | --- |
| Sex (Male) | | | | 136 (68.0%) | |
| Age (Mean±SD) | | | | 62.7±11.0 | |
| Hypertension | | | | 167 (83.5%) | |
| Diabetes | | | | 64 (32.0%) | |
| Hyperlipidemia | | | | 123 (61.5%) | |
| Coronary heart disease | | | | 28 (14.0%) | |
| Smoking | | | | 94 (47.0%) | |
| Hyperhomocysteinemia | | | | 61 (30.5%) | |
| Chronic Kidney Disease | | | | 17 (8.5%) | |
| Family history of stroke | | | | 70 (35.0%) | |
| Drugs | | antiplatelet | | 67 (33.5%) | |
|  |  | anticoagulant | | 2 (1.0%) | |
| Presence of WMH | | | | 185(92.5%) | |
| Severe WMH | | | | 97(52.4%) | |
| Distribution of WMH | | Frontal region | | 177(95.7%) | |
|  |  | Parietooccipital region | | 143(77.3%) | |
|  |  | Temporal region | | 78(42.2%) | |
|  |  | Infratentorial | | 38(20.5%) | |
|  |  | Basal ganglia | | 81(43.8%) | |
| Presence of LI | | | | 157(78.5%) | |
| Multiple LI | | | | 132(84.1%) | |
| Distribution of LI | | Frontal region | | 103(65.6%) | |
|  |  | Parietooccipital region | | 32(20.4%) | |
|  |  | Temporal region | | 27(17.2%) | |
|  |  | Infratentorial | | 47(29.9%) | |
|  |  | Basal ganglia | | 125(79.6%) | |
|  |  | Cerebellum | | 8(5.1%) | |
|  |  | Brain stem | | 39(24.8%) | |
| Presence of CMB | | | | 45(32.6%) | |
| Severe CMB | | | | 23(51.1%) | |
| Distribution of CMB | | Lobe | | 28(62.2%) | |
|  |  | Deep | | 33(73.3%) | |
|  |  | Infratentorial | | 21(46.7%) | |

Abbreviation: WMH=White Matter Hyperintensities, LI=Lacunar Infarction; CMB=Cerebral Microbleeds.
